# The impact of 10-valent pneumococcal conjugate vaccine on the incidence of admissions to hospital with hypoxaemic and non-hypoxaemic pneumonia in Kenyan children

**DOI:** 10.1371/journal.pgph.0004888

**Published:** 2025-07-28

**Authors:** Ilsa L. Haeusler, E. Wangeci Kagucia, Christian Bottomley, Mark Otiende, Joyce Nyiro, J. Anthony G. Scott

**Affiliations:** 1 Department of Infectious Disease Epidemiology, Faculty of Epidemiology and Population Health, London School of Hygiene & Tropical Medicine, London, United Kingdom; 2 Institute for Global Health, University College London, London, United Kingdom; 3 KEMRI-Wellcome Trust Research Programme, Kilifi, Kenya; Qatar University College of Medicine, QATAR

## Abstract

Observational evidence suggests that pneumococcal conjugate vaccines (PCVs) are more effective at reducing the incidence of hypoxaemic pneumonia compared to non-hypoxaemic pneumonia. We examined the impact of 10-valent PCV (PCV10, Synflorix) on hypoxaemic pneumonia hospital admissions using data from an existing PCV impact study of clinical and radiographic pneumonia. PCV10 was introduced, with catch-up among children under 5 years, in the Kilifi Health and Demographic Surveillance System (KHDSS) in January 2011. We undertook an interrupted time-series (ITS) analysis using seasonally-adjusted segmented Poisson regression of hypoxaemic and non-hypoxaemic pneumonia, accounting for secular trends, from January 2007 to December 2019 among KHDSS residents aged 2–59 months. The median monthly crude incidence of hypoxaemic pneumonia per 100,000 person-years was 95 (IQR 80–139) in the pre-PCV10 period. Time-series regression estimated PCV10 introduction was associated with an increase in the incidence of hypoxaemic pneumonia in children aged 2–59 months (IRR 1.63, 95% CI 1.10–2.41), whereas it was associated with a decrease in the incidence of non-hypoxaemic pneumonia (IRR 0.61, 95% CI 0.48–0.77). Despite the consistent pneumonia surveillance and robust ITS analysis which accounted for pre-PCV10 secular trend and seasonality, the apparent association with hypoxaemic pneumonia is likely due to unmeasured time-varying confounders around the time of vaccine introduction, such as the epidemiology of other respiratory pathogens. This study highlights limitations in the analysis and interpretation of observational data in vaccine impact studies against pneumonia.

## Introduction

Pneumococcal conjugate vaccines (PCVs) were first licenced in 2000 and are currently in programmatic use in 166 countries, 47 of which are supported by Gavi, The Vaccine Alliance [1,2]. Whilst these vaccines have contributed to the reduction in disease caused by *Streptococcus pneumoniae*, pneumococcal pneumonia remains an important cause of morbidity and mortality globally [3,4].

Accurately quantifying PCV effectiveness and impact for the range of important pneumonia outcomes is complicated by low positive predictive values (PPV) of pneumonia diagnoses for pneumococcal pneumonia, particularly of clinically diagnosed pneumonia. In an effort to increase the PPV of clinically diagnosed pneumonia for pneumococcal pneumonia, pneumonia with hypoxaemia is increasingly used as an outcome. Impact and effectiveness estimates for hypoxaemic pneumonia have been reported following programmatic introduction of PCV in Malawi, The Gambia, Papua New Guinea, Laos, Mongolia and Fiji [5–10]. In each of these studies, the positive impact or effectiveness estimates for hypoxaemic pneumonia are larger than those for clinical pneumonia.

PCV10 (Synflorix) was introduced into the Kenyan immunisation programme in 2011. Demographic and hospital admission surveillance data have been collected in Kilifi, Kenya since 2002 [11]. Previous analyses from this population estimated that in children under 5 years, the introduction of PCV10 was associated with a reduction in clinically and radiologically-diagnosed pneumonia of 27% (95% CI 3–46) and 48% (95% CI 14–68), respectively [12]. In this study, we report the impact of PCV10 on hypoxaemic pneumonia using additional data from existing pneumonia surveillance in Kilifi.

## Materials and methods

### Ethics statement

Parents or guardians provided written consent for inclusion. This study was approved by the Kenya Medical Research Institute Scientific and Ethics Review Unit, Oxford Tropical Research Ethics Committee, and the London School of Hygiene and Tropical Medicine Ethics Committee.

### Surveillance and population

The Kilifi Health and Demographic Surveillance System (KHDSS) operates within Kilifi County, Kenya [11,13]. Household surveys record births, deaths and migration events 4–6 monthly. After steady population growth to its maximum in 2012, the KHDSS population remained static at approximately 44,000 for children aged 2–59 months (S1 Fig). The Kilifi County Hospital (KCH) offers the main inpatient paediatric facility serving the KHDSS population [14]. On admission to KCH, KHDSS and clinical data are linked by matching a set of demographic details [11].

PCV10 was introduced into the Kenyan routine immunisation programme in 2011 with a 3 + 0 schedule, administered at 6, 10 and 14 weeks of life. A national catch-up campaign offered three doses to infants during 2011. In Kilifi County, two additional catch-up campaigns between January to the end of March 2011 offered two doses to children aged 12–59 months. In 2011, 79.9% and 85.5% of children aged 2–11 months and 12–23 months had at least two doses of PCV10, respectively. These proportions increased to 84.3% and 88.6% in 2016 [12,15].

### Outcomes

Pneumonia was defined according to syndromic definitions as per the 2005 WHO guidelines [16]. Hypoxaemia is one clinical sign which defines very severe pneumonia (so all hypoxaemic pneumonia cases were classified as very severe). Hypoxaemia was defined as a blood oxygen saturation of <90% on admission (recorded at sea level using Edan, SPECTRO2 and Nellcor pulse oximeter devices). Non-hypoxaemic pneumonia was defined as pneumonia without hypoxaemia on admission. Only oxygen saturations recorded at the time of admission were used to classify a case as hypoxaemic or non-hypoxaemic.

All children were screened on admission for malaria (defined as the presence of any parasitaemia on thick and thin Giemsa-stained blood slides) and severe malnutrition (classified as a binary variable, defined as weight-for-age less than −3 Z scores below the median of the WHO standards) [17]. Since 2007, HIV was tested for in children whose parents or guardians gave verbal consent. At clinician discretion, among children with respiratory symptoms, nasopharyngeal samples were tested for respiratory syncytial virus (RSV) by PCR. Chest x-rays were taken for investigation of severe or very severe pneumonia from April 2006 [12].

## Statistical analysis

Data were accessed on 15/03/2021. Data were available from May 2002 to December 2019. There was a large decline in the incidence of WHO-defined clinical pneumonia between May 2002 and December 2006 (likely due to a decline in malaria which can cause similar clinical features to pneumonia resulting in possible misclassification), so the analysis was restricted from January 2007 to December 2019 [18].

Between January 2007 and December 2019, monthly incidence rates of hypoxaemic and non-hypoxaemic pneumonia in children aged ≥2 to ≤59 months were calculated by dividing the monthly case count by the mid-monthly population and reported per 100,000. KHDSS mid-monthly populations were used to estimate monthly person years at risk. The case counts were restricted to admissions to KCH by KHDSS residents.

An interrupted time-series (ITS) analysis was conducted using segmented Poisson regression. A step-change model (without a lag) was hypothesised *a priori* because PCV10 was introduced with a catch-up campaign for all children under 5 years of age, achieving good coverage as described under ‘surveillance and population’. Hypoxaemic and non-hypoxaemic pneumonia were modelled separately. Monthly case counts were included as the response variable with the log mid-monthly KHDSS population included as an offset variable to model rates. A linear time-trend was included and seasonality was accounted for by including calendar month as a categorical variable. The analysis excluded months during which vaccine roll out took place (January to March 2011) and months during which healthcare workers were on strike (S1 Table) [12,19,20]. Autocorrelation and heteroscedasticity were accounted for by adjusting the standard errors using the Newey-West method up to lag 3 [20]. Wald p-values were used throughout.

Model checking was conducted by plotting the deviance residuals over time and by plotting the partial autocorrelation function of the deviance residuals. For both hypoxaemic and non-hypoxaemic pneumonia, there was evidence of remaining autocorrelation (lag 1). Residual autocorrelation and heteroscedasticity were accounted for by adjusting the standard errors using the Newey-West method up to lag 3 [21]. Wald p-values were used throughout.

A stratified analysis was conducted to investigate whether the effect of PCV10 on the incidence rate of hypoxaemic and non-hypoxaemic pneumonia varied by age group (2–11 months, 12–23 months, 24–59 months), and chest x-ray, RSV and malaria results. Analyses were performed in Stata version 18.

### Role of the funding source

The funders had no role in any aspect of the development of this study.

## Results

**Fig 1:**
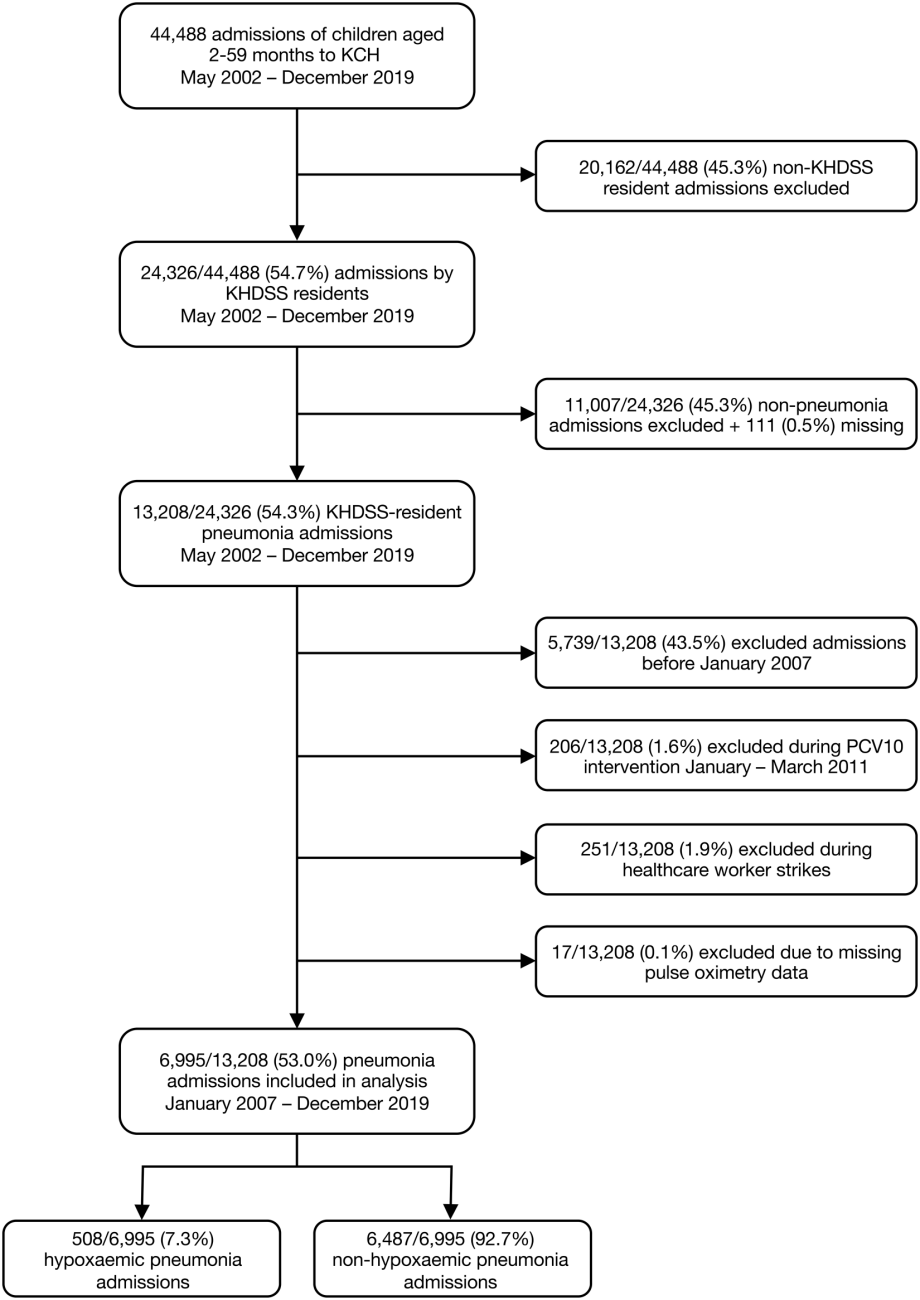
Flow diagram of admissions to Kilifi County Hospital (KCH). KHDSS = Kilifi Health and Demographic Surveillance System.

Between May 2002 and December 2019, there were 24,326 admissions to KCH of KHDSS-resident children aged 2–59 months (Fig 1). Of these, 13,208 (54.3%) were admitted with WHO-defined clinical pneumonia. Following exclusions, the total number of pneumonia cases was 6,995 (53.0%), of which 6,487 (92.7%) were non-hypoxaemic and 508 (7.3%) were hypoxaemic.

Of the 24,326 admissions between May 2002 and December 2019, 111 (0.5%) were excluded due to missing pneumonia symptoms (cough and difficulty breathing). Seventeen (of 13,208, 0.1%) pneumonia admissions were missing pulse oximetry data. Among pneumonia admissions, 808/6,995 (11.6%) were missing HIV test data, 196/6,995 (2.8%) were missing malnutrition data, 317/6,995 (4.5%) were missing malaria data, 4,545/6,995 (65.0%) were missing chest x-ray data, and 3,296/6,995 (47.1%) were missing RSV data.

Children with hypoxaemic pneumonia were younger than those with non-hypoxaemic pneumonia (median age 11.5 months (IQR 5–23) and 14 months (IQR 7–27), respectively), and a larger proportion was HIV-positive and malnourished (S2 Table). A larger proportion of non-hypoxaemic admissions was fully PCV10 vaccinated compared to hypoxaemic admissions: 2,3462/3,109 (75.5%) and 214/318 (67.3%) respectively.

The incidence rate of all-cause admissions declined until 2013 then stabilised at approximately 2,000 per 100,000 person-years for children aged 2–59 months (S2 Fig). Mortality rates following all-cause admissions (KHDSS residents who died in hospital from any cause from the KHDSS population) and pneumonia admissions were stable during the included study period at 0–200 per 100,000 person-years per month (S3 Fig and S4 Fig). Among children aged 2–59 months, the annual percentage of pneumonia admissions that were resident in the KHDSS decreased from a maximum of 62.5% in 2004 to a minimum of 42.1% in 2019 (S5 Fig for monthly values).

There was no evidence of a time-varying association between the incidence of admission to hospital with pneumonia (hypoxaemic and non-hypoxaemic) and age (S6 Fig) or sex (S7 Fig). There was no evidence of a change in proportion of children who had co-morbid malnutrition with hypoxaemic or non-hypoxaemic pneumonia over time (S8 Fig), nor co-morbid HIV with hypoxaemic or non-hypoxaemic pneumonia (from mid-2005 onwards, S9 Fig). The incidence rate of malaria was high and rapidly decreased prior to 2007, thereafter remaining relatively stable (S10 Fig). For RSV, the rate slightly increased until 2008/2009, thereafter it decreased (S11 Fig).

The crude median monthly incidence rate per 100,000 person-years for children aged 2–59 months admitted with hypoxaemic pneumonia was 95 (IQR 80–139) in the pre-intervention period and 79 (IQR 53–131) in the post-intervention period (Table 1). On visual inspection of the trend, the incidence increased between 2011 and 2014, and subsequently decreased to less than pre-intervention levels (S12 Fig). Although the crude annual incidence rates decreased over the study period, the rates fluctuated between years (S3 Table). For non-hypoxaemic pneumonia, the pre-intervention crude median monthly incidence rate per 100,000 person-years was 1,791 (IQR 1,363–2,360) and 834 (IQR 673–993) in the post-intervention period (Table 1). There was fluctuation with an overall reduction in the rate of non-hypoxaemic pneumonia over the surveillance period in each age group (S3 Table and S13 Fig).

**Table 1:** Crude median monthly counts and incidence rates of hypoxaemic and non-hypoxaemic pneumonia in KHDSS-resident children admitted to Kilifi County Hospital during the pre- and post-intervention period, by age group.

Age (months)	Case counts				Incidence rate per 100,000			
	Pre-intervention		Post-intervention		Pre-intervention		Post-intervention	
	Median	IQR	Median	IQR	Median	IQR	Median	IQR
**Hypoxaemic pneumonia**								
2-59	3.5	3–5	3	2–5	95	80–139	79	53–131
2-11	2	1–3	1	0–2	325	159–487	164	0–329
12-23	1	0–1	1	0–1	120	0–132	123	0–132
24-59	1	0–2	0	0–1	44	0–89	0	0–43
**Non-hypoxaemic pneumonia**								
2-59	65.5	49.5–87.5	31	25–37	1,791	1,363–2,360	834	673–993
2-11	25.5	19.5–36.5	12	9–17.5	3,930	3,234–5,701	1,867	1,450–2,798
12-23	20	13.5–27	9	6–12	2,557	1,726–3,404	1,175	779–1,570
24-59	19	14.5–24	9	7–13	858	654–1,056	382	303–556

Based on 48 and 96 pre- and post-intervention time-points respectively (January 2007-December 2019). Excluding 3-month intervention period and 9 months of healthworker strikes. Pneumonia as defined by WHO 2005 definition. Hypoxaemic pneumonia defined as pneumonia with oxygen saturations on admission of <90%. IQR = interquartile range; KHDSS = Kilifi Health and Demographic Surveillance System.

**Fig 2:**
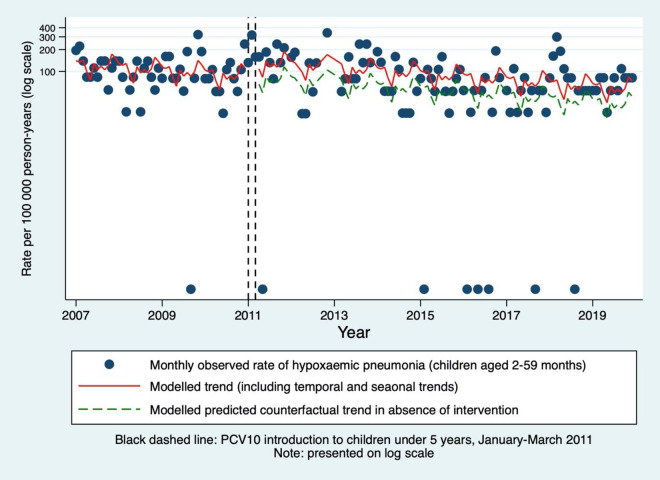
Monthly incidence rates of hypoxaemic pneumonia in KHDSS-resident children aged 2-59 months admitted to Kilifi County Hospital. Predictions using segmented Poisson model, accounting for time and seasonality, excluding PCV10 introduction and healthworker strikes. Hypoxaemic pneumonia defined as pneumonia with oxygen saturations on admission of <90%. KHDSS = Kilifi Health and Demographic Surveillance System.

The segmented Poisson model, adjusted for time-trend and seasonality through calendar month, estimated a 63% increase in the incidence of hypoxaemic pneumonia associated with PCV10 introduction in children aged 2–59 months (IRR 1.63, 95% CI 1.10–2.41, p = 0.014; Table 2 and Fig 2). Although the effect estimate increased with increasing age group, there was no statistical evidence of a difference between strata (on the basis of overlapping confidence intervals) and no evidence of effect in individual age strata. The same model estimated a 1% reduction per month of the baseline incidence of hypoxaemic pneumonia, following adjustment for seasonality and PCV10 introduction (IRR 0.992, 95% CI 0.987–0.997, p = 0.001; S4 Table). Given the apparent increase in incidence between 2011–2014, a *post-hoc* sensitivity analysis censored these years (the pre- and post-intervention years were 2007–2010 and 2015–2019, respectively, as the incidence appeared more stable between 2015 and 2019). This estimated a 37% reduction in hypoxaemic pneumonia associated with PCV10 introduction in children aged 2–59 months (IRR 0.63, 95% CI 0.30–1.33, p = 0.225).

**Table 2:** Summary regression model estimates for KHDSS-resident children admitted to Kilifi County Hospital with hypoxaemic and non-hypoxaemic pneumonia by age group. Fitted by segmented Poisson regression, adjusted for time-trend and seasonality (through calendar month). Newey-West standard errors used to account for autocorrelation (lag three). All estimates based on 144 time points (48 pre, 96 post): data between January 2007 and December 2019, excluding 9 months of healthworker strikes and 3 months of intervention roll-out. Pneumonia as defined by WHO 2005 definition. Hypoxaemic pneumonia defined as pneumonia with oxygen saturations on admission of <90%. P-values are two-sided (Wald p-values). IRR = incidence rate ratio; KHDSS = Kilifi Health and Demographic Surveillance System.

Age (months)	Hypoxaemic pneumonia			Non-hypoxaemic pneumonia		
	IRR	95% CI	p-value	IRR	95% CI	p-value
2-59	1.63	1.10–2.41	0.014	0.61	0.48–0.77	<0.001
2-11	1.49	0.89–2.48	0.126	0.66	0.50–0.87	0.004
12-23	1.76	0.87–3.57	0.116	0.65	0.49–0.87	0.004
24-59	1.87	0.81–4.31	0.140	0.50	0.37–0.69	<0.001

**Fig 3:**
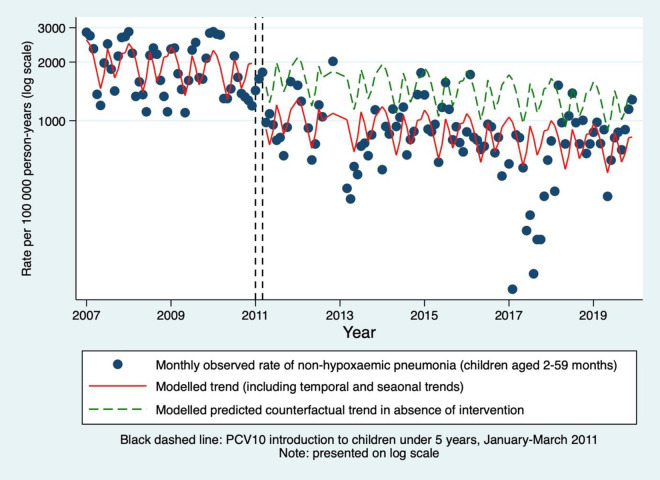
Monthly incidence rates of non-hypoxaemic pneumonia in KHDSS-resident children aged 2-59 months admitted to Kilifi County Hospital. Predictions using segmented Poisson model, accounting for time and seasonality, excluding PCV10 introduction and healthworker strikes. Hypoxaemic pneumonia defined as pneumonia with oxygen saturations on admission of <90%. KHDSS = Kilifi Health and Demographic Surveillance System.

The equivalent model for non-hypoxaemic pneumonia estimated a decrease in its incidence by 39% associated with PCV10 introduction (IRR 0.61, 95% CI 0.48–0.77, p < 0.001; Table 2 and Fig 3). Although the largest effect was in the 24–59 age group (IRR 0.50, 95% CI 0.39–0.67, p < 0.001), there was no statistical evidence of a difference in estimates between strata (Table 2). After adjusting for season and PCV10 introduction, the monthly baseline incidence of non-hypoxaemic pneumonia decreased by 1% per month (IRR 0.997, 95% CI 0.994–0.999, p = 0.015; S4 Table).

Seasonality was incorporated through inclusion of calendar month because there was evidence of confounding by seasonality (S5 Table). Compared with seasonal adjustment through Fourier terms, calendar month adjustment led to an improved model fit (S5 Table). There was no evidence of interaction between study time and vaccine period for either hypoxaemic or non-hypoxaemic pneumonia (IRR 1.00, 95% CI 0.99–1.00, p = 0.373 and 1.00, 0.99–1.00, p = 0.705 respectively). This suggests there was no evidence that the trend was different during the pre- and post-intervention periods.

Separate models were built to stratify hypoxaemic and non-hypoxaemic pneumonia estimates by chest x-ray (radiological evidence of pneumonia or no radiological evidence of pneumonia), RSV (positive or negative), and malaria (parasitaemia positive or negative; S6 Table). The stratified hypoxaemic model estimated that there was a large effect in RSV-positive hypoxaemic pneumonia associated with PCV10 introduction in children aged 2–59 months (IRR 4.31, 95% CI 1.06–17.56, p = 0.041) compared to RSV negative (IRR 1.85, 95% CI 1.15–2.98, p = 0.012). For malaria, there was no evidence of an effect of vaccine introduction in hypoxaemic and non-hypoxaemic pneumonia cases which were malaria positive (IRR 1.39, 95% CI 0.44–4.44, p = 0.578 and IRR 0.94, 95% CI 0.48–1.83, p = 0.840, respectively), whereas the effect sizes in those who were malaria negative were similar to unstratified estimates. However, for each stratified analysis, overlapping confidence intervals suggest there was no evidence of any difference between strata.

## Discussion

These results suggest that the introduction of PCV10 to children under five years in Kilifi was temporally associated with a decrease in the incidence of non-hypoxaemic all-cause pneumonia by 39% in 2–59-month-olds. In contrast, PCV10 introduction was temporally associated with a 63% increase in the incidence of hypoxaemic all-cause pneumonia. The scatter trend indicated an increase in incidence from 2011 to 2014, giving the appearance of a multiyear ‘epidemic’ of hypoxaemic pneumonia around the time of vaccine introduction, with a subsequent reduction to less than pre-intervention levels. Excluding these unstable years from the analysis estimated a non-significant reduction of 37% in hypoxaemic pneumonia in children aged 2–59 months. All-cause hypoxaemic pneumonia incidence declined during the surveillance period by 1% per month following adjustment for seasonality and PCV10.

The cumulative body of evidence from Kilifi supports a reduction in pneumococcal disease following PCV10 introduction across multiple outcomes: PCV10-type carriage declined (age-standardised adjusted prevalence ratio 0.26) [20]; PCV10-type invasive disease declined in vaccinated age groups (adjusted IRR 0.08) as well as unvaccinated age groups [20]; invasive disease caused by any serotype declined (adjusted IRR 0.32); and clinical and x-ray confirmed pneumonia declined (adjusted IRR 0.73 and 0.52, respectively) [12]. A systematic review of PCV10 and PCV13 impact studies in children aged under 5 years identified a reduction of 7–60% in all-cause pneumonia hospital admissions, 8–90% in severe pneumonia, 12–79% in radiological pneumonia, and 45–85% in pneumococcal pneumonia [22]. This highlights the large variability in impact estimates by pneumonia endpoint in different populations.

Our findings are not consistent with existing evidence on hypoxaemic pneumonia. An ITS analysis estimated the introduction of PCV13 in Malawi was associated with an increase in the incidence of pneumonia in children aged under 5 years by 47% whereas hypoxaemic pneumonia incidence decreased by 47% [5]. This analysis was by ‘early’ and ‘late’ post-PCV13 time-periods, so the time-trend was estimated from post-intervention data. The strength of evidence of effect was weaker for hospitalised hypoxaemic cases, a population more similar to our analysis, with a non-significant reduction of 36%. In The Gambia, a before/after analysis with adjustment for rate of referral estimated that hypoxaemic pneumonia declined by 60% in the 2–59 month age group, with the greatest effect of a reduction of 69% in the 12–23 month age group [6]. *Streptococcus pneumoniae* was isolated from 168/801 (21.0%) and 337/2,1954 (1.5%) of hypoxic and clinical pneumonia cases, respectively.

A test-negative case/control study in children aged under 5 in Laos estimated PCV13 effectiveness was 37% against hypoxaemia among patients with pneumonia [8]. A Papua New Guinean study adopted the same design [7]. PCV13 effectiveness against hypoxaemia among patients with pneumonia was 28.7%. These results suggest that pneumococcal disease is likely concentrated in more severe pneumonia, and that pneumococcal pneumonias are more likely than alternative aetiologies to be hypoxaemic.

Comparing pre-PCV13 and post-PCV13 periods using negative binomial regression accounting for secular trends and district effect, a study from Mongolia found there was no reduction in all-cause clinical pneumonia or hypoxaemic pneumonia in children aged 2–59 months (adjusted IRR 1.01 and 0.83, respectively) [9]. Finally, a synthetic control ITS study from Fiji estimated PCV10 introduction was associated with a reduction in hypoxaemic pneumonia of 46% in children aged 2–23 months [10].

There is no biologically plausible explanation for a causal association between PCV10 introduction and the increase in hypoxaemic pneumonia. The non-hypoxaemic result is in line with previous analyses from Kilifi and other populations, providing reassurance of the internal validity of the findings. Despite the robust surveillance data and ITS analysis used in this study, it is likely that residual confounding explains the hypoxaemic pneumonia result. In an attempt to identify a possible source of confounding, stratified analyses were conducted by malaria parasitaemia results. The signs and symptoms of malaria can be difficult to differentiate from pneumonia and may be misdiagnosed as such [18]. There was no evidence that malaria accounted for the findings as PCV10 introduction remained associated with an increase in hypoxaemic pneumonia cases which were malaria negative.

RSV accounts for approximately one third of hospital admissions with pneumonia [23]. Over the course of the surveillance period, there was no evidence of an increase in the rate of RSV positive admissions around the time of vaccine introduction. However, there was a large proportion of missing RSV data in pneumonia cases and the biases influencing RSV testing are unknown. It is likely that sicker children were more likely to be tested as the amount of missing data in hypoxaemic cases was 22.8% compared to 49.0% of non-hypoxaemic cases. If RSV was under-reported around the time of PCV10 introduction to 2014, it is possible that the epidemic behaviour of RSV could account for the increase in hypoxaemic pneumonia incidence. There was a weak suggestion that RSV may in part account for the apparent association, as the stratum-specific estimate for RSV positive hypoxaemic pneumonia was very large (4.31, 95% CI 1.06–17.56, p = 0.041) compared to RSV negative (1.85, 95% CI 1.15–2.98, p = 0.012). However there remains a positive effect in RSV negative hypoxaemic pneumonia which has not been explained.

There are other possible hypotheses to explain the apparent association. It is possible that a multiyear outbreak of an undiagnosed virus (or viruses) could explain the findings if infection tended to cause pneumonia with hypoxaemia. The vaccination campaign may have caused a change in health-seeking behaviour because of having been vaccinated, whereby children present to hospital later with more severe disease, accounting for both an increase in hypoxaemic pneumonia incidence and a decrease in non-hypoxaemic pneumonia incidence. Healthworker strikes, between 2011 and 2013, are temporally coincident with PCV10 introduction and these may have caused residents to be more reluctant to attend hospital during this period and some may have delayed their presentation, resulting in the same effect. If a delay in presentation accounts for the increase in hypoxaemic pneumonia incidence, an increase in the mortality rate following admission with pneumonia might be expected (all else remaining constant). However, the mortality rate of KHDSS-resident children admitted with pneumonia decreased until 2010 and thereafter remained stable. Improvement in access to care in the form of transport and road infrastructure may have resulted in more severely unwell children presenting to hospital who otherwise would have died in the community. To account for the apparent association, these phenomena would need to be temporally associated with vaccine introduction, and not accounted for by the time-trend. The PERCH study, done just as PCV10 was introduced and lasting two years, involved rigorous testing and healthworker training including on pulse oximetry [23]. This may have increased the sensitivity of oximetry testing to detecting hypoxaemia.

Hypoxaemic pneumonia has gained traction as an outcome because of the hypothesis that hypoxaemia increases the PPV of clinical pneumonia for pneumococcal pneumonia. There is evidence that *S. pneumoniae* is associated with an increased tendency to cause hypoxaemia over other pathogens [7,8]. There is also evidence that bacteria are more common aetiological pathogens in very severe pneumonia (of which hypoxaemia is one sign) compared with severe pneumonia, so the PPV for bacterial pneumonia (of all causes) could be increased through severity [23]. However, hypoxaemia can be caused by a range of other bacterial pathogens, viruses and malaria [23] in addition to other diseases including anaemia and cardiac disease which can be difficult to differentiate from pneumonia or may co-exist. Another explanation is that the efficacy of PCVs varies with pneumonia severity. If PCVs prevent severe disease more effectively that mild disease, a greater impact against hypoxaemic pneumonia than non-hypoxaemic pneumonia would be expected. If PCVs reduce severity of disease, a reduction in hypoxaemic pneumonia may be accompanied by an increase in non-hypoxaemic pneumonia. These scenarios were not observed in this study.

To conclude, this analysis identified a 63% increase in hypoxaemic pneumonia in children aged 2–59 months which was temporally associated with the introduction of PCV10 in Kilifi. This is likely driven by uncontrolled confounding, such as changes in the epidemiology of other respiratory pathogens, and is very unlikely to be attributable to PCV10 introduction. Confidence in this conclusion is drawn from the study’s prospective cohort design, which included longitudinal surveillance of multiple disease endpoints and confounders, and strong evidence of impact against other pneumococcal disease endpoints. This study therefore highlights important limitations in the analysis and interpretation of observational data in pneumococcal pneumonia impact studies.

## Supporting information

S1 Fig Mid-year population estimates of children aged 2–59 months resident in the Kilifi Health and Demographic Surveillance System, 2002–2019. (DOCX)

S2 Fig Monthly rate of all-cause admissions by Kilifi Demographic Surveillance System residents to Kilifi County Hospital, by age group, May 2002 to December 2019. (DOCX)

S3 Fig Monthly mortality rate following all-cause admissions by Kilifi Demographic Surveillance System residents to Kilifi County Hospital, by age group, May 2002 to December 2019. (DOCX)

S4 Fig Monthly mortality rate following admission with pneumonia by Kilifi Demographic Surveillance System residents to Kilifi County Hospital, by age group, May 2002 to December 2019. (DOCX)

S5 Fig Monthly percent of pneumonia admissions which were resident in the Kilifi Health and Demographic Surveillance System, by age group, May 2002 to December 2019. (DOCX)

S6 Fig Median age of monthly admissions to Kilifi County Hospital with hypoxaemic and non-hypoxaemic pneumonia by Kilifi Health and Demographic Surveillance System residents aged 2–59 months, May 2002 to December 2019. Hypoxaemic pneumonia defined as pneumonia with oxygen saturations on admission of <90%.
(DOCX)

S7 Fig Monthly proportion of admissions by males to Kilifi County Hospital with hypoxaemic and non-hypoxaemic pneumonia by Kilifi Health and Demographic Surveillance System residents aged 2–59 months, May 2002 to December 2019. Hypoxaemic pneumonia defined as pneumonia with oxygen saturations on admission of <90%.
(DOCX)

S8 Fig Monthly proportion of hypoxaemic and non-hypoxaemic pneumonia admissions with co-morbid severe malnutrition by Kilifi Health and Demographic Surveillance System residents aged 2–59 months to Kilifi County Hospital, May 2002 to December 2019. Hypoxaemic pneumonia defined as pneumonia with oxygen saturations on admission of <90%.
(DOCX)

S9 Fig Monthly proportion of hypoxaemic and non-hypoxaemic pneumonia admissions with co-morbid HIV by Kilifi Health and Demographic Surveillance System residents aged 2–59 months to Kilifi County Hospital, May 2002 to December 2019. Hypoxaemic pneumonia defined as pneumonia with oxygen saturations on admission of <90%.
(DOCX)

S10 Fig Monthly incidence rate of admissions with malaria to Kilifi County Hospital by Kilifi Health and Demographic Surveillance System residents aged 2–59 months, May 2002 to December 2019. (DOCX)

S11 Fig Monthly incidence rate of admissions with Respiratory Syncytial Virus to Kilifi County Hospital by Kilifi Health and Demographic Surveillance System residents aged 2–59 months, May 2002 to December 2019. (DOCX)

S12 Fig Monthly incidence rate of hypoxaemic pneumonia admissions to Kilifi County Hospital by Kilifi Health and Demographic Surveillance System residents, by age group, January 2007 to December 2019. Hypoxaemic pneumonia defined as pneumonia with oxygen saturations on admission of <90%.
(DOCX)

S13 Fig Monthly incidence rate of non-hypoxaemic pneumonia admissions to Kilifi County Hospital by Kilifi Health and Demographic Surveillance System residents, by age group, January 2007 to December 2019. Non-hypoxaemic pneumonia defined as pneumonia with oxygen saturations on admission of ≥90%.
(DOCX)

S1 Table Dates of healthcare worker strikes at Kilifi County Hospital. (DOCX)

S2 Table Characteristics of Kilifi Health and Demographic Surveillance System-resident children admitted to Kilifi County Hospital with hypoxaemic and non-hypoxaemic pneumonia. (DOCX)

S3 Table Crude annual incidence rates of hypoxaemic and non-hypoxaemic pneumonia in Kilifi Health and Demographic Surveillance System-resident children admitted to Kilifi County Hospital between 2002 and 2019, by age group. (DOCX)

S4 Table Incidence rate ratios (PCV10 introduction, time trend and calendar months) for hypoxaemic and non-hypoxaemic pneumonia. (DOCX)

S5 Table Incidence rate ratios (PCV10 introduction) for hypoxaemic and non-hypoxaemic pneumonia using different methods of accounting for seasonality. (DOCX)

S6 Table Incidence rate ratios (PCV10 introduction) for hypoxaemic and non-hypoxaemic pneumonia, stratified by chest x-ray, RSV and malaria results. (DOCX)

## References

[1] Goldblatt D, Miller E. Pneumococcal pneumonia. Thorax. 2020;75(1):6–7. doi: 10.1136/thoraxjnl-2019-214135 31753962 PMC6929703

[2] International Vaccine Access Center (IVAC) Johns Hopkins Bloomberg School of Public Health. Pneumococal Conjugate Vacccine (PCV) Introduction & Use. Available from: https://view-hub.org/vaccine/pcv

[3] Liu L, Oza S, Hogan D, Chu Y, Perin J, Zhu J, et al. Global, regional, and national causes of under-5 mortality in 2000-15: an updated systematic analysis with implications for the Sustainable Development Goals. Lancet. 2016;388(10063):3027–35. doi: 10.1016/S0140-6736(16)31593-8 27839855 PMC5161777

[4] You D, Hug L, Ejdemyr S, Idele P, Hogan D, Mathers C, et al. Global, regional, and national levels and trends in under-5 mortality between 1990 and 2015, with scenario-based projections to 2030: a systematic analysis by the UN Inter-agency Group for Child Mortality Estimation. Lancet. 2015;386(10010):2275–86. doi: 10.1016/S0140-6736(15)00120-8 26361942

[5] McCollum ED, Nambiar B, Deula R, Zadutsa B, Bondo A, King C, et al. Impact of the 13-Valent Pneumococcal Conjugate Vaccine on Clinical and Hypoxemic Childhood Pneumonia over Three Years in Central Malawi: An Observational Study. PLoS One. 2017;12(1):e0168209. doi: 10.1371/journal.pone.0168209 28052071 PMC5215454

[6] Gambia Pneumococcal Surveillance Group, Mackenzie GA, Hill PC, Jeffries DJ, Ndiaye M, Sahito SM, et al. Impact of the introduction of pneumococcal conjugate vaccination on invasive pneumococcal disease and pneumonia in The Gambia: 10 years of population-based surveillance. Lancet Infect Dis. 2021;21(9):1293–302. doi: 10.1016/S1473-3099(20)30880-X 34280357 PMC8384632

[7] Blyth CC, Britton KJ, Nguyen CD, Sapura J, Kave J, Nivio B, et al. Effectiveness of 13-valent pneumococcal conjugate vaccine against hypoxic pneumonia and hospitalisation in Eastern Highlands Province, Papua New Guinea: An observational cohort study. Lancet Reg Health West Pac. 2022;22:100432. doi: 10.1016/j.lanwpc.2022.100432 35308576 PMC8927990

[8] Weaver R, Nguyen CD, Chan J, Vilivong K, Lai JYR, Lim R, et al. The effectiveness of the 13-valent pneumococcal conjugate vaccine against hypoxic pneumonia in children in Lao People’s Democratic Republic: An observational hospital-based test-negative study. Lancet Reg Health West Pac. 2020;2:100014. doi: 10.1016/j.lanwpc.2020.100014 34327372 PMC8315332

[9] von Mollendorf C, Ulziibayar M, Nguyen CD, Batsaikhan P, Suuri B, Luvsantseren D, et al. Effect of Pneumococcal Conjugate Vaccine on Pneumonia Incidence Rates among Children 2-59 Months of Age, Mongolia, 2015-2021. Emerg Infect Dis. 2024;30(3):490–8. 38407131 10.3201/eid3003.230864 PMC10902538

[10] Reyburn R, Tuivaga E, Nguyen CD, Ratu FT, Nand D, Kado J, et al. Effect of ten-valent pneumococcal conjugate vaccine introduction on pneumonia hospital admissions in Fiji: a time-series analysis. Lancet Glob Health. 2021;9(1):e91–8. doi: 10.1016/S2214-109X(20)30421-6 33227258

[11] Scott JAG, Bauni E, Moisi JC, Ojal J, Gatakaa H, Nyundo C, et al. Profile: The Kilifi Health and Demographic Surveillance System (KHDSS). Int J Epidemiol. 2012;41(3):650–7. doi: 10.1093/ije/dys062 22544844 PMC3396317

[12] Silaba M, Ooko M, Bottomley C, Sande J, Benamore R, Park K, et al. Effect of 10-valent pneumococcal conjugate vaccine on the incidence of radiologically-confirmed pneumonia and clinically-defined pneumonia in Kenyan children: an interrupted time-series analysis. Lancet Glob Health. 2019;7(3):e337–46. doi: 10.1016/S2214-109X(18)30491-1 30784634 PMC6379823

[13] Otieno GP, Bottomley C, Khagayi S, Adetifa I, Ngama M, Omore R, et al. Impact of the Introduction of Rotavirus Vaccine on Hospital Admissions for Diarrhea Among Children in Kenya: A Controlled Interrupted Time-Series Analysis. Clin Infect Dis. 2020;70(11):2306–13. doi: 10.1093/cid/ciz912 31544211 PMC7245159

[14] Scott JAG, Berkley JA, Mwangi I, Ochola L, Uyoga S, Macharia A, et al. Relation between falciparum malaria and bacteraemia in Kenyan children: a population-based, case-control study and a longitudinal study. Lancet. 2011;378(9799):1316–23. doi: 10.1016/S0140-6736(11)60888-X 21903251 PMC3192903

[15] Adetifa IMO, Karia B, Mutuku A, Bwanaali T, Makumi A, Wafula J, et al. Coverage and timeliness of vaccination and the validity of routine estimates: Insights from a vaccine registry in Kenya. Vaccine. 2018;36(52):7965–74. doi: 10.1016/j.vaccine.2018.11.005 30416017 PMC6288063

[16] World Health Organization. Pocket book of hospital care for children: guidelines for the management of common childhood illnesses. 2005. Available from: http://apps.who.int/iris/bitstream/handle/10665/43206/9241546700.pdf?sequence=1 24006557

[17] World Health Organization. WHO child growth standards: length/height-for-age, weight-for-age, weight-for-length, weight-for-height and body mass index-for-age: methods and development. 2006. Available from: https://www.who.int/publications/i/item/924154693X

[18] Bottomley C, Kamau A, Awori JO, Driscoll AJ, Park DE, Sow SO. Misclassification of malaria as pneumonia in children in sub-Saharan Africa. Int J Epidemiol. 2025;54(2). 10.1093/ije/dyaf040 PMC11984459 40209071

[19] Ong’ayo G, Ooko M, Wang’ondu R, Bottomley C, Nyaguara A, Tsofa BK, et al. Effect of strikes by health workers on mortality between 2010 and 2016 in Kilifi, Kenya: a population-based cohort analysis. Lancet Glob Health. 2019;7(7):e961–7. doi: 10.1016/S2214-109X(19)30188-3 31129126 PMC6560003

[20] Hammitt LL, Etyang AO, Morpeth SC, Ojal J, Mutuku A, Mturi N, et al. Effect of ten-valent pneumococcal conjugate vaccine on invasive pneumococcal disease and nasopharyngeal carriage in Kenya: a longitudinal surveillance study. Lancet. 2019;393(10186):2146–54. doi: 10.1016/S0140-6736(18)33005-8 31000194 PMC6548991

[21] Wooldridge JM. Chapter 12. Introductory Econometrics: A Modern Approach. 4th ed. South-Western Cengage Learning. 2009.

[22] Reyburn R, Tsatsaronis A, von Mollendorf C, Mulholland K, Russell FM, ARI Review group. Systematic review on the impact of the pneumococcal conjugate vaccine ten valent (PCV10) or thirteen valent (PCV13) on all-cause, radiologically confirmed and severe pneumonia hospitalisation rates and pneumonia mortality in children 0-9 years old. J Glob Health. 2023;13:05002. doi: 10.7189/jogh.13.05002 36734192 PMC9896304

[23] O’Brien KL, Baggett HC, Brooks WA, Feikin DR, Hammitt LL, Higdon MM, et al. Causes of severe pneumonia requiring hospital admission in children without HIV infection from Africa and Asia: the PERCH multi-country case-control study. Lancet. 2019;394(10200):757–79. 31257127 10.1016/S0140-6736(19)30721-4 PMC6727070

