# Supplementary material for: The impact of 10-valent pneumococcal conjugate vaccine on the incidence of admissions to hospital with hypoxaemic and non-hypoxaemic pneumonia in Kenyan children
Source: PLOS Glob Public Health. 2025 Jul 28;5(7):e0004888. doi: 10.1371/journal.pgph.0004888 (PMC12303342; doi:10.1371/journal.pgph.0004888)
Supplement: S1 Fig — (DOCX) [file pgph.0004888.s001.docx]

S1 Fig: Mid-year population estimates of children aged 2-59 months resident in the Kilifi Health and Demographic Surveillance System, 2002-2019.
