# Supplementary material for: The impact of 10-valent pneumococcal conjugate vaccine on the incidence of admissions to hospital with hypoxaemic and non-hypoxaemic pneumonia in Kenyan children
Source: PLOS Glob Public Health. 2025 Jul 28;5(7):e0004888. doi: 10.1371/journal.pgph.0004888 (PMC12303342; doi:10.1371/journal.pgph.0004888)
Supplement: S3 Fig — (DOCX) [file pgph.0004888.s003.docx]

S3 Fig: Monthly mortality rate following all-cause admissions by Kilifi Demographic Surveillance System residents to Kilifi County Hospital, by age group, May 2002 to December 2019.
