# Supplementary material for: The impact of 10-valent pneumococcal conjugate vaccine on the incidence of admissions to hospital with hypoxaemic and non-hypoxaemic pneumonia in Kenyan children
Source: PLOS Glob Public Health. 2025 Jul 28;5(7):e0004888. doi: 10.1371/journal.pgph.0004888 (PMC12303342; doi:10.1371/journal.pgph.0004888)
Supplement: S12 Fig — Hypoxaemic pneumonia defined as pneumonia with oxygen saturations on admission of <90%. (DOCX) [file pgph.0004888.s012.docx]

S12 Fig: Monthly incidence rate of hypoxaemic pneumonia admissions to Kilifi County Hospital by Kilifi Health and Demographic Surveillance System residents, by age group, January 2007 to December 2019. Hypoxaemic pneumonia defined as pneumonia with oxygen saturations
