# Supplementary material for: The impact of 10-valent pneumococcal conjugate vaccine on the incidence of admissions to hospital with hypoxaemic and non-hypoxaemic pneumonia in Kenyan children
Source: PLOS Glob Public Health. 2025 Jul 28;5(7):e0004888. doi: 10.1371/journal.pgph.0004888 (PMC12303342; doi:10.1371/journal.pgph.0004888)
Supplement: S13 Fig — Non-hypoxaemic pneumonia defined as pneumonia with oxygen saturations on admission of ≥90%. (DOCX) [file pgph.0004888.s013.docx]

S13 Fig: Monthly incidence rate of non-hypoxaemic pneumonia admissions to Kilifi County Hospital by Kilifi Health and Demographic Surveillance System residents, by age group, January 2007 to December 2019. Non-hypoxaemic pneumonia defined as pneumonia with oxygen saturations on admission of ≥90%.
