# Supplementary material for: The impact of 10-valent pneumococcal conjugate vaccine on the incidence of admissions to hospital with hypoxaemic and non-hypoxaemic pneumonia in Kenyan children
Source: PLOS Glob Public Health. 2025 Jul 28;5(7):e0004888. doi: 10.1371/journal.pgph.0004888 (PMC12303342; doi:10.1371/journal.pgph.0004888)
Supplement: S1 Table — (DOCX) [file pgph.0004888.s014.docx]

S1 Table: Dates of healthcare worker strikes at Kilifi County Hospital.

| **Dates** | **Number of days** | **Staff involved** |
| --- | --- | --- |
| 5 December 2011 - 13 December 2011 | 9 | Doctors |
| 1 March 2012 - 15 March 2012 | 15 | Nurses |
| 13 September 2012 - 4 October 2012 | 23 | Doctors |
| 2 December 2012 - 22 December 2012 | 22 | Nurses |
| 16 January 2013 - 11 February 2013 | 27 | Nurses |
| 10 December 2013 - 23 December 2013 | 14 | Doctors, nurses |
| 5 December 2016 - 31 December 2016 | 27 | Undocumented |
| Strikes affecting paediatric wards during the surveillance period (2002-2019). | | |
