# Supplementary material for: The impact of 10-valent pneumococcal conjugate vaccine on the incidence of admissions to hospital with hypoxaemic and non-hypoxaemic pneumonia in Kenyan children
Source: PLOS Glob Public Health. 2025 Jul 28;5(7):e0004888. doi: 10.1371/journal.pgph.0004888 (PMC12303342; doi:10.1371/journal.pgph.0004888)
Supplement: S2 Table — (DOCX) [file pgph.0004888.s015.docx]

S2 Table: Characteristics of KHDSS-resident children admitted to Kilifi County Hospital with hypoxaemic and non-hypoxaemic pneumonia.

|  | **Hypoxaemic pneumonia** | | **Non-hypoxaemic pneumonia** | |
| --- | --- | --- | --- | --- |
|  | (n=508) | | (n=6,487) | |
|  | n | % | n | % |
| Age |  |  |  |  |
| 2-11 months | 254 | 50.0 | 2,697 | 41.6 |
| 12-23 months | 134 | 26.4 | 1,876 | 28.9 |
| 24-59 months | 120 | 23.6 | 1,914 | 29.5 |
| Sex (male) | 259 | 51.0 | 3,647 | 56.2 |
| Died | 100 | 19.7 | 214 | 3.3 |
| HIV-positive | 34 | 6.7 | 278 | 4.3 |
| Malnourished | 164 | 32.3 | 1,166 | 18.0 |

Admitted between January 2007-December 2019 (excluding cases with missing pulse oximetry data and admissions during the 3-month intervention period and 9 months of healthworker strikes). Hypoxaemic pneumonia defined as pneumonia with oxygen saturations on admission of <90%. KHDSS = Kilifi Health and Demographic Surveillance System.
