# Supplementary material for: The impact of 10-valent pneumococcal conjugate vaccine on the incidence of admissions to hospital with hypoxaemic and non-hypoxaemic pneumonia in Kenyan children
Source: PLOS Glob Public Health. 2025 Jul 28;5(7):e0004888. doi: 10.1371/journal.pgph.0004888 (PMC12303342; doi:10.1371/journal.pgph.0004888)
Supplement: S3 Table — (DOCX) [file pgph.0004888.s016.docx]

| *S15 Table:* Crude annual incidence rates of hypoxaemic and non-hypoxaemic pneumonia in KHDSS-resident children admitted to Kilifi County Hospital between 2002 and 2019, by age group. | | | | | | | | | | | | | | | | | | | | |  |
| --- | --- | --- | --- | --- | --- | --- | --- | --- | --- | --- | --- | --- | --- | --- | --- | --- | --- | --- | --- | --- | --- |
| **Age (months)** | **Year** | | | | | | | | | | | | | | | | | | | |  |
|  | 2002* | 2003 | 2004 | 2005 | 2006 | 2007 | 2008 | 2009 | 2010 | 2011 | 2012 | 2013 | 2014 | 2015 | 2016 | 2017 | 2018 | 2019 |  |  |  |
| **Hypoxaemic pneumonia (incidence rate per 100,000 person years)** | | | | | | | | | | | | | | | | | | | | | |
| 2-59 | 143 | 195 | 225 | 153 | 213 | 125 | 88 | 125 | 93 | 154 | 135 | 128 | 85 | 75 | 57 | 48 | 102 | 70 |  |  |  |
| 2-11 | 296 | 486 | 702 | 465 | 690 | 339 | 292 | 298 | 378 | 397 | 513 | 392 | 214 | 198 | 222 | 151 | 316 | 161 |  |  |  |
| 12-23 | 237 | 191 | 214 | 208 | 114 | 125 | 77 | 166 | 81 | 203 | 191 | 216 | 87 | 133 | 55 | 55 | 130 | 129 |  |  |  |
| 24-59 | 70 | 113 | 97 | 47 | 101 | 65 | 34 | 59 | 25 | 70 | 14 | 31 | 47 | 22 | 15 | 18 | 36 | 26 |  |  |  |
| **Non-hypoxaemic pneumonia (incidence rate per 100,000 person years)** | | | | | | | | | | | | | | | | | | | | | |
| 2-59 | 2,337 | 3,573 | 3,339 | 2,372 | 2,534 | 2,143 | 1,770 | 1,968 | 1,787 | 1,155 | 1,039 | 582 | 1,010 | 982 | 821 | 439 | 925 | 856 |  |  |  |
| 2-11 | 4,527 | 7,704 | 8,103 | 6,180 | 6,590 | 5,267 | 4,527 | 4,648 | 4,251 | 3,016 | 2,656 | 1,459 | 2,585 | 2,221 | 1,996 | 1,206 | 2,115 | 2,216 |  |  |  |
| 12-23 | 3,548 | 4,991 | 4,464 | 3,234 | 3,259 | 3,220 | 2,271 | 2,736 | 2,256 | 1,547 | 1,507 | 906 | 1,100 | 1,376 | 1,190 | 518 | 1,141 | 1,185 |  |  |  |
| 24-59 | 1,329 | 1,928 | 1,617 | 1,019 | 1,053 | 865 | 817 | 898 | 997 | 506 | 439 | 251 | 532 | 507 | 390 | 210 | 541 | 374 |  |  |  |
| *Surveillance began in May 2002. All other years are complete. PCV10 introduction and strike periods are included. Pneumonia as defined by WHO 2005 definition. Hypoxaemic pneumonia defined as pneumonia with oxygen saturations on admission of <90%. KHDSS = Kilifi Health and Demographic Surveillance System. | | | | | | | | | | | | | | | | | | | |  |  |
