# Supplementary material for: The impact of 10-valent pneumococcal conjugate vaccine on the incidence of admissions to hospital with hypoxaemic and non-hypoxaemic pneumonia in Kenyan children
Source: PLOS Glob Public Health. 2025 Jul 28;5(7):e0004888. doi: 10.1371/journal.pgph.0004888 (PMC12303342; doi:10.1371/journal.pgph.0004888)
Supplement: S4 Table — (DOCX) [file pgph.0004888.s017.docx]

S4 Table: Incidence rate ratios (PCV10 introduction, time trend and calendar months) for hypoxaemic and non-hypoxaemic pneumonia.

|  | **Hypoxaemic pneumonia** | | | **Non-hypoxaemic pneumonia** | | |
| --- | --- | --- | --- | --- | --- | --- |
|  | IRR | 95% CI | p-value | IRR | 95% CI | p-value |
| **PCV10 introduction** | 1.63 | 1.10–2.41 | 0.014 | 0.61 | 0.48–0.77 | <0.001 |
| **Time (months)** | 0.992 | 0.987–0.997 | 0.001 | 0.997 | 0.994–0.999 | 0.015 |
| **Season** |  |  |  |  |  |  |
| January | Base |  |  | Base |  |  |
| February | 0.96 | 0.69–1.35 | 0.820 | 0.95 | 0.80–1.12 | 0.522 |
| March | 1.03 | 0.62–1.71 | 0.908 | 0.83 | 0.66–1.04 | 0.110 |
| April | 0.73 | 0.49–1.09 | 0.124 | 0.68 | 0.56–0.83 | <0.001 |
| May | 0.56 | 0.36–0.88 | 0.012 | 0.57 | 0.48–0.68 | <0.001 |
| June | 0.95 | 0.73–1.25 | 0.732 | 0.68 | 0.56–0.82 | <0.001 |
| July | 0.79 | 0.57–1.09 | 0.150 | 0.92 | 0.77–1.10 | 0.355 |
| August | 0.89 | 0.60–1.34 | 0.577 | 0.81 | 0.67–0.98 | 0.027 |
| September | 0.80 | 0.50–1.26 | 0.337 | 0.66 | 0.54–0.79 | <0.001 |
| October | 0.92 | 0.61–1.37 | 0.672 | 0.73 | 0.62–0.86 | <0.001 |
| November | 1.34 | 0.92–1.96 | 0.131 | 0.88 | 0.71–1.10 | 0.256 |
| December | 1.18 | 0.80–1.75 | 0.403 | 0.89 | 0.69–1.16 | 0.384 |

Residents of the Kilifi Health and Demographic Surveillance System aged 2-59 months admitted to Kilifi County Hospital. Fitted by segmented Poisson regression, adjusted for time-trend and seasonality (through calendar month). Newey-West standard errors used to account for autocorrelation (lag three). All estimates based on 144 time points (48 pre, 96 post): data between January 2007 and December 2019, excluding 9 months of healthworker strikes and 3 months of intervention roll-out. Pneumonia as defined by WHO 2005 definition. Hypoxaemic pneumonia defined as pneumonia with oxygen saturations on admission of <90%. P-values are two-sided (Wald p-values). IRR = incidence rate ratio.
