# Supplementary material for: The impact of 10-valent pneumococcal conjugate vaccine on the incidence of admissions to hospital with hypoxaemic and non-hypoxaemic pneumonia in Kenyan children
Source: PLOS Glob Public Health. 2025 Jul 28;5(7):e0004888. doi: 10.1371/journal.pgph.0004888 (PMC12303342; doi:10.1371/journal.pgph.0004888)
Supplement: S5 Table — (DOCX) [file pgph.0004888.s018.docx]

S5 Table: Incidence rate ratios (PCV10 introduction) for hypoxaemic and non-hypoxaemic pneumonia using different methods of accounting for seasonality.

| **Adjustment method** | **IRR** | **95% CI** | **p-value** | **AIC** | **Deviance** |
| --- | --- | --- | --- | --- | --- |
| **Hypoxaemic pneumonia** | |  |  |  |  |
| No seasonal adjustment | 1.49 | 0.95–2.34 | 0.081 | 4.379 | 211.726 |
| Calendar month | 1.63 | 1.10–2.41 | 0.014 | 4.367 | 188.042 |
| 1 sine/cosine pairs | 1.62 | 1.09–2.42 | 0.018 | 4.321 | 199.334 |
| 2 sine/cosine pairs | 1.62 | 1.09–2.43 | 0.018 | 4.340 | 198.064 |
| 3 sine/cosine pairs | 1.63 | 1.10–2.41 | 0.015 | 4.330 | 192.626 |
| **Non-hypoxaemic pneumonia** | | |  |  |  |
| No seasonal adjustment | 0.59 | 0.44–0.78 | <0.001 | 10.862 | 765.818 |
| Calendar month | 0.61 | 0.48–0.77 | <0.001 | 9.807 | 591.984 |
| 1 sine/cosine pairs | 0.61 | 0.47–0.79 | <0.001 | 10.480 | 706.904 |
| 2 sine/cosine pairs | 0.61 | 0.48–0.78 | <0.001 | 9.984 | 631.411 |
| 3 sine/cosine pairs | 0.62 | 0.48–0.79 | <0.001 | 9.800 | 600.574 |

Residents of the Kilifi Health and Demographic Surveillance System aged 2-59 months admitted to Kilifi County Hospital. Fitted by segmented Poisson regression, adjusted for time-trend and seasonality (through calendar month). Newey-West standard errors used to account for autocorrelation (lag three). All estimates based on 144 time points (48 pre, 96 post): data between January 2007 and December 2019, excluding 9 months of healthworker strikes and 3 months of intervention roll-out. Pneumonia as defined by WHO 2005 definition. Hypoxaemic pneumonia defined as pneumonia with oxygen saturations on admission of <90%. P-values are two-sided (Wald p-values). IRR = incidence rate ratio.
