# Supplementary material for: The impact of 10-valent pneumococcal conjugate vaccine on the incidence of admissions to hospital with hypoxaemic and non-hypoxaemic pneumonia in Kenyan children
Source: PLOS Glob Public Health. 2025 Jul 28;5(7):e0004888. doi: 10.1371/journal.pgph.0004888 (PMC12303342; doi:10.1371/journal.pgph.0004888)
Supplement: S6 Table — (DOCX) [file pgph.0004888.s019.docx]

| *S6 Table:* Incidence rate ratios (PCV10 introduction) for hypoxaemic and non-hypoxaemic pneumonia, stratified by chest x-ray, RSV and malaria results. | | | | | | | | | |
| --- | --- | --- | --- | --- | --- | --- | --- | --- | --- |
|  |  | **Hypoxaemic pneumonia** | | | | **Non-hypoxaemic pneumonia** | | | |
|  |  | **n (%)** | **IRR** | **95% CI** | **p-value** | **n (%)** | **IRR** | **95% CI** | **p-value** |
| **Chest x-ray** | Pneumonia | 93 (18.3) | 3.34 | 1.36–8.22 | 0.009 | 439 (6.8) | 0.61 | 0.36–1.03 | 0.070 |
|  | No pneumonia | 163 (32.1) | 4.38 | 2.08–9.25 | <0.001 | 1,755 (27.1) | 0.99 | 0.60–1.62 | 0.970 |
|  | Missing | 252 (49.6) |  |  |  | 4,293 (66.2) |  |  |  |
| **RSV** | Positive | 57 (11.2) | 4.31 | 1.06–17.56 | 0.041 | 718 (11.1) | 1.19 | 0.53–2.64 | 0.680 |
|  | Negative | 335 (65.9) | 1.85 | 1.15–2.98 | 0.012 | 2,589 (39.9) | 1.11 | 0.87–1.43 | 0.400 |
|  | Missing | 116 (22.8) |  |  |  | 3,180 (49.0) |  |  |  |
| **Malaria** | Positive | 34 (6.7) | 1.39 | 0.44–4.44 | 0.578 | 639 (9.9) | 0.94 | 0.48–1.83 | 0.840 |
|  | Negative | 462 (90.9) | 1.65 | 1.08–2.51 | 0.021 | 5,543 (85.5) | 0.60 | 0.47–0.75 | <0.001 |
|  | Missing | 12 (2.4) |  |  |  | 305 (4.7) |  |  |  |
| Residents of the Kilifi Health and Demographic Surveillance System aged 2-59 months admitted to Kilifi County Hospital. Fitted by segmented Poisson regression, adjusted for time-trend and seasonality (through calendar month). Newey-West standard errors used to account for autocorrelation (lag three). All estimates based on 144 time points (48 pre, 96 post): data between January 2007 and December 2019, excluding 9 months of healthworker strikes and 3 months of intervention roll-out. Pneumonia as defined by WHO 2005 definition. Hypoxaemic pneumonia defined as pneumonia with oxygen saturations on admission of <90%. P-values are two-sided (Wald p-values). IRR = incidence rate ratio; RSV = Respiratory Syncytial Virus. | | | | | | | | | |
